# FreeSASA: An open source C library for solvent accessible surface area calculations

**DOI:** 10.12688/f1000research.7931.1

**Published:** 2016-02-18

**Authors:** Simon Mitternacht

**Affiliations:** 1 University Library, University of Bergen, Bergen, Norway

**Keywords:** Structural Bioinformatics, Solvent Accessible Surface Area, Command-line tool, C library, Python module

## Abstract

Calculating solvent accessible surface areas (SASA) is a run-of-the-mill calculation in structural biology. Although there are many programs available for this calculation, there are no free-standing, open-source tools designed for easy tool-chain integration. FreeSASA is an open source C library for SASA calculations that provides both command-line and Python interfaces in addition to its C API. The library implements both Lee and Richards’ and Shrake and Rupley’s approximations, and is highly configurable to allow the user to control molecular parameters, accuracy and output granularity. It only depends on standard C libraries and should therefore be easy to compile and install on any platform. The library is well-documented, stable and efficient. The command-line interface can easily replace closed source legacy programs, with comparable or better accuracy and speed, and with some added functionality.

## Introduction

Exposing apolar molecules to water is highly unfavorable, and minimizing the hydrophobic free energy is an important driving force in the folding of macromolecules ( Finkelstein & Ptitsyn, 2002). The solvent accessible surface area (SASA) of a molecule gives a measure of the contact area between molecule and solvent. Although the exact quantitative relation between surface area and free energy is elusive, the SASA can be used to compare different molecules or different conformations of the same molecule, or for example measure the surface that is buried due to oligomerization.

To define the SASA, let a spherical probe represent a solvent molecule. Roll the probe over the surface of a larger molecule. The surface area traced by the center of the probe is the SASA of the larger molecule ( Lee & Richards, 1971). Two classical approximations are commonly used to calculate SASA: one by Lee and Richards (L&R) where the surface is approximated by the outline of a set of slices ( 1971), and one by Shrake & Rupley (1973) (S&R) where the surface of each sphere is approximated by a set of test points. The SASA can be calculated to arbitrary precision by refining the resolution of both. The surface area can also be calculated analytically ( Fraczkiewicz & Braun, 1998), which is useful when the gradient is needed, or by various other approximations, tailored for different purposes ( Cavallo *et al.*, 2003; Drechsel *et al.*, 2014; Sanner *et al.*, 1996; Weiser *et al.*, 1999; Xu & Zhang, 2009).

There are many tools available to calculate SASA. The most popular program for command line use is probably NACCESS ( Hubbard & Thornton, 1993) (freely available for academic use), which is an efficient Fortran implementation of the L&R approximation. Another well-known command line tool is DSSP, which primarily calculates the secondary structure and hydrogen bonds of a protein structure, but provides the SASA as well ( Touw *et al.*, 2015) (using S&R, open source). There are also some web services available, for example Getarea, which calculates the surface analytically ( Fraczkiewicz & Braun, 1998), and Triforce which uses a semianalytical tessellation approach ( Drechsel *et al.*, 2014) (also available for command line use). In addition, most molecular dynamics simulation packages include tools to analyze SASA from trajectories.

FreeSASA is intended to fill the same niche as NACCESS, and a number of other similar programs: a simple and fast command-line tool that “does one thing and does it well” and can be easily integrated into tool chains. The advantage of FreeSASA is that it is open source (GNU General Public License 3), and provides both C and Python APIs in addition to its command line interface. It has sensible default parameters and no obligatory configuration for casual users (the only required input is a structure), but also allows full control over all calculation parameters. Dependencies have been kept to a minimum: compilation only requires standard C and GNU libraries. The library is thread-safe, and some effort has gone into dealing gracefully with various errors. The code ships with thorough documentation, which is also available online at http://freesasa.github.io/doxygen/. Although functionality and availability have been the primary motivating factors for writing this library, performance tests show that FreeSASA is as fast as or faster than legacy programs when run on a single CPU core. In addition, a large portion of the calculation has been parallelized, which gives significant additional speed when run on multicore processors.

## Methods

### Implementation

***Calculations.*** Both S&R and L&R are pretty straightforward to implement, and both require first determining which atoms are in contact, and then calculating the overlap between each atom and its neighbors. Finding contacts is done using cell lists, which means the contact calculation is an *O*( *N*) operation. Both algorithms then treat each atom independently, making also the second part of the calculation *O*( *N*). In addition, this second part is trivially parallelizable.

For L&R, instead of slicing the whole protein in one go, each atom is sliced individually. The L&R calculation is thus parameterized by the number of slices per atom, i.e. small atoms have thinner slices than large atoms.

The Fibonacci spiral gives a good approximation to an even distribution of points on the sphere ( Swinbank & Purser, 2006), allowing efficient generation of an arbitrary number of S&R test points. The cell lists provide the first of the two lattices in the double cubic lattice optimization for this algorithm ( Eisenhaber *et al.*, 1995), the second lattice (for the test points) is not implemented in FreeSASA, for now.

The correctness of the implementations was tested by first inspecting the surfaces visually. In the two atom case, results were verified against analytical calculations. Another verification came from comparing the results of high precision SASA calculations using the two independent algorithms. In addition, using the L&R algorithm gives identical results to NACCESS when the same resolution and atomic radii are used.

***Radius assignment.*** An important step of the calculation is assigning a radius to each atom. The default in FreeSASA is to use the *ProtOr* radii by Tsai *et al.* (1999). The library recognizes the 20 standard amino acids (plus Sec and Pyl), and the standard nucleotides (plus a few nonstandard ones). Tsai *et al.* do not mention phosphorus and selenium; these atoms are assigned a radius of 1.8 and 1.9 Å respectively.

By default, hydrogen atoms and HETATM records are ignored in Protein Data Bank (PDB) files. If included, the library recognizes three common HETATM entries: the acetyl and NH _2_ capping groups, and water, and assigns ProtOr radii to these. Otherwise the van der Waals radius of the element is used, taken from the paper by Mantina *et al.* (2009). For elements outside of the 44 main group elements treated by Mantina *et al.*, or if completely different radii are desired, users can provide their own configuration.

Users can specify their own atomic radii either through the API or by providing a configuration file. The library ships with a few sample configuration files, including one that provides a subset of the NACCESS parameterization, and one with the default ProtOr parameters. In addition, scripts are provided to automatically generate ProtOr configurations from PDB CONECT entries, such as those in the Chemical Component Dictionary ( Westbrook *et al.*, 2015). These can then be appended to the default configuration.

### Operation

Building the FreeSASA library and command-line interface only requires standard C and GNU libraries and a C99-compliant compiler, and should be straightforward on any UNIX system (has been tested in Mac OS X 10.8 and Debian 8), and not too difficult in Windows (not tested). Building the Python bindings requires Cython (tested with version 0.21). The library ships with an Autotools build configuration, but the source itself is simple enough to be possible to compile “manually”, if necessary.

***Command-line interface.*** Building FreeSASA creates the binary freesasa. The simplest program call, with default parameters, is

  $ freesasa 3wbm.pdb
                    


using the structure with PDB code 3wbm as an example (a protein-RNA complex). The above produces the following output

  ## freesasa 1.0.1 ##
                    


  PARAMETERS
  algorithm    : Lee & Richards
  probe-radius : 1.400
  threads      : 2
  slices       : 20
                    


  INPUT
  source  : 3wbm.pdb
  chains  : ABCDXY
  atoms   : 3714
                    


  RESULTS (A^2)
  Total   : 25190.77
  Apolar  : 11552.38
  Polar   : 13638.39
  CHAIN A :  3785.49
  CHAIN B :  4342.33
  CHAIN C :  3961.12
  CHAIN D :  4904.30
  CHAIN X :  4156.46
  CHAIN Y :  4041.08
                    


The numbers in the results section are the SASA values (in Å ^2^) for the respective groups of atoms.

As an illustration of a few of the other configuration options, and how to use the program as a PDB file filter, the command

  $ freesasa -n 100 --print-as-B-values --no-log < 3wbm.pdb > 3wbm.sasa
                    


calculates the SASA of a PDB-file passed via stdin, using 100 slices per atom. The flag --no-log suppresses the regular output. The output will instead, because of the flag --print-as-B-values, be the provided PDB-file with the SASA of each atom replacing the temperature factors, and the atomic radii stored in the occupancy factor field.

By calling with the option --chain-groups,

  $ freesasa --chain-groups=ABCD+XY 3wbm.pdb
                    


two calculations are appended to the original output, one where only the four chains A, B, C and D have been included, and one with only X and Y.

The option --select can be used to select a set of atoms using a subset of the selection syntax used in the program Pymol ( DeLano, 2002). For example, the command

  $ freesasa --select="RNA, resn A+U+G+C"
                    


will produce the following output (after the regular output shown above)

  SELECTIONS
  RNA :    8197.53
                    


where *RNA* is simply the user-defined name of the selection, and the number the contribution to the total SASA from the bases A, U, G and C (which we in this particular case could have got by simply adding the areas for the chains X and Y in the sample output above).

The command

  $ freesasa -h
                    


prints a help message listing all available options, including other ways to redirect output and how to change different calculation parameters (the most detailed information can be found online at http://freesasa.github.io/doxygen/CLI.html).

***C API.*** The C code below illustrates how to perform a SASA-calculation on the same PDB-file as above, using the C API, with default parameters. The functions and types used are all defined in the header freesasa.h.

**Figure 1.**
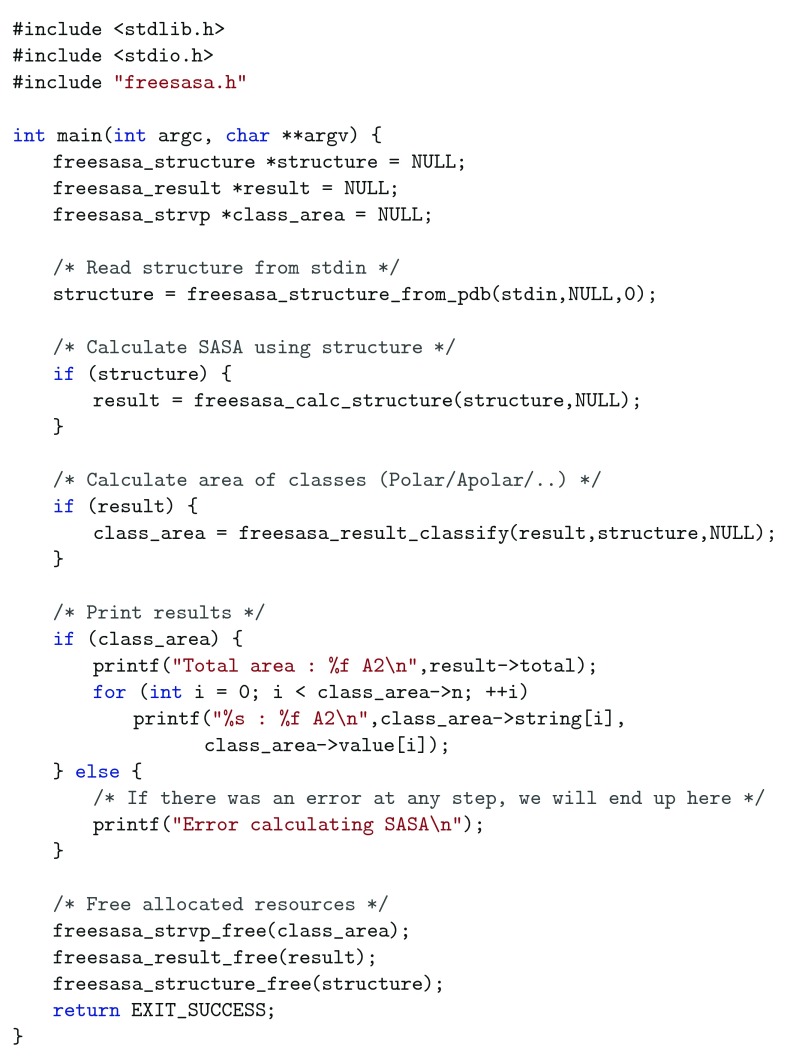
C API. Illustration of how to use the C API including rudimentary error handling.

  FILE *fp = fopen("3wbm.pdb","r");
  freesasa_structure *structure = freesasa_structure_from_pdb(fp, NULL, 0);
  freesasa_result *result = freesasa_calc_structure(structure, NULL);
  printf("Total area : %f A2\n", result->total);
                    


The two points where null pointers are passed as arguments are places where atom classifiers and calculation parameters could have been provided. A more elaborate example that includes error handling and freeing of allocated resources can be seen in Figure 1.

The API also allows the user to calculate the SASA of a set of coordinates with associated radii. The code below puts two atoms at positions ${\vec{x}}_{1}=(1,1,1)$ and ${\vec{x}}_{2}=(2,2,2)$ with radii *r*_1_ = 2 and *r*_2_ = 3, respectively, and outputs the SASA of the individual atoms.

  double coord[] = {1.0, 1.0, 1.0, 2.0, 2.0, 2.0};
  double radius[] = {2.0, 3.0};
  freesasa_result *result = freesasa_calc_coord(coord, radius, 2, NULL);
  printf("A1 = %f, A2 = %f\n", result->sasa[0], result->sasa[1]);
                    


***Python API.*** The library includes Python bindings that export most of the C API to Python. The Python code below gives the same output as the example in Figure 1. Error handling is excluded for brevity.

  import freesasa
                    


  structure = freesasa.Structure("3wbm.pdb")
  result = freesasa.calc(structure)
  classArea = freesasa.classifyResults(result,structure)
                    


  print "Total : %.2f A2" % result.totalArea()
  for key in classArea:
    print key, ": %.2f A2" % classArea[key]
                    


## Results

List of PDB codes used for the performance analysis This set was generated from the most restrictive list of structures in the PISCES database ( Wang & Dunbrack, 2003). 88 PDB files were selected randomly from a set of size intervals in this list, to get an approximately even distribution in size. Click here for additional data file. Copyright: © 2016 Mitternacht S 2016 Data associated with the article are available under the terms of the Creative Commons Zero "No rights reserved" data waiver (CC0 1.0 Public domain dedication).

**Figure 2.**
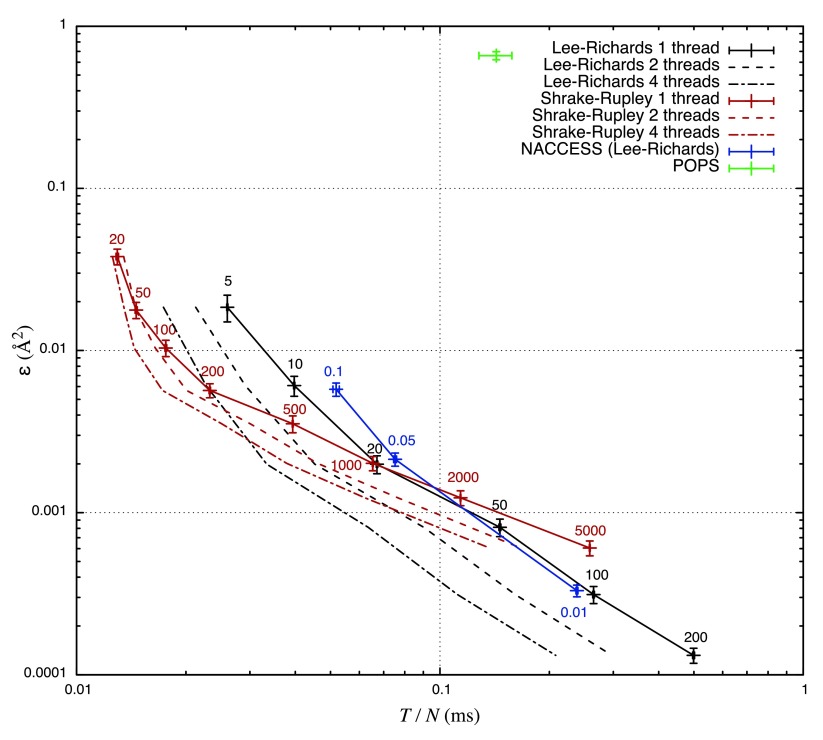
Precision and calculation time. The mean of the error *ε* in SASA vs *T*/ *N*, for the two algorithms in FreeSASA plus the programs NACCESS and POPS. Labels indicate the resolution used for each set of calculations, and error bars the standard error along both axes. The solid lines are only there to guide the eye, and the dashed lines indicate the analogous lines when using 2 and 4 threads in FreeSASA. An L&R run with 1000 slices was used as *A*_ref_ when calculating *ε* for both approximations. NACCESS uses L&R and was run with three values of the z-parameter (0.1, 0.05 and 0.01, corresponding to 10, 20 and 100 slices per atom), a run with z-parameter 0.005 was used as *A*_ref_ (using even lower z-values gave inconsistent results). The NACCESS reference calculation was also used as reference for POPS. All programs were compiled using GCC 4.9.3 with the optimization flag “-Ofast” and the tests were run on an Intel Core i5-2415M CPU at 2.30 GHz. The raw data for this figure can be found in Dataset 2.

**Figure 3.**
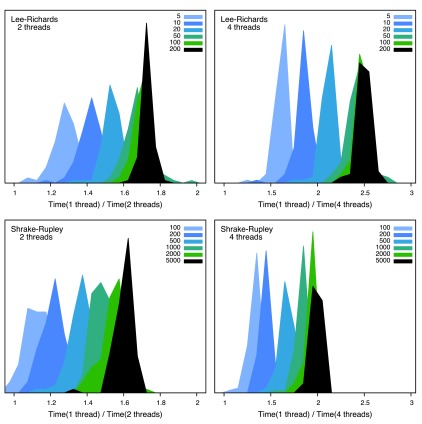
Parallelization. The histograms shows the distribution of the calculation time using two or four threads divided by the time using one thread. Thus if this fraction is two or four, respectively, we have “perfect” parallelization. The legends indicate the resolution of the calculation: for L&R, slices per atom, and for S&R, number of test points. The raw data for this figure can be found in Dataset 2.

Zip-archive with raw data for the performance analysis in Figure 2 and Figure 3 See the file explanation.txt for an overview of the contents of the archive. Click here for additional data file. Copyright: © 2016 Mitternacht S 2016 Data associated with the article are available under the terms of the Creative Commons Zero "No rights reserved" data waiver (CC0 1.0 Public domain dedication).

The computational efficiency of the two algorithms was compared by running the FreeSASA command-line program with different parameters on a set of 88 PDB structures selected from the PISCES database ( Wang & Dunbrack, 2003) (see Dataset 1 for a list). PISCES specifies a specific chain in each structure, but in the following all chains were used, which resulted in the largest structure having over 30,000 atoms (1jz8). To average out some variation in the running time in these rather short calculations (in some cases < 10 ms), the fastest calculations were run two to five times. As we will see below, error bars are relatively small along that axis.

To measure the accuracy of the two algorithms, a reference SASA value, *A*_ref_, was calculated using L&R with 1000 slices per atom for each structure. The error of a given SASA-value, *A*, is then *ε* = | *A*– *A*_ref_|/ *N*, where *N* is the number of atoms in the structure. Calculation time *T* is measured as the wall time of the entire calculation including reading and writing files. Dataset 2 contains the values of *A*, *A*_ref_, *N* and *T* used to produce Figure 2 and Figure 3.

Figure 2 shows *ε* versus *T*/ *N*, averaged over the 88 PDB structures. At low resolution S&R is considerably faster than L&R, and at high resolution L&R is faster, with a crossover at around 1000 test points or 20 slices per atom (20 slices is the default setting in FreeSASA).

Comparisons were done with NACCESS 2.1.1 ( Hubbard & Thornton, 1993), DSSP 2.2.1 ( Touw *et al.*, 2015), NSOL 1.7 ( Masuya, 2003), POPS 1.6.4 ( Cavallo *et al.*, 2003) and Triforce 0.1 ( Drechsel *et al.*, 2014). The list could potentially have been a lot longer; some programs were left out on the basis of being closed source, poorly documented or no longer available. NACCESS was included in spite of its limiting license due to its popularity. The SASA facilities in molecular dynamics packages were not considered since these cater to a different use case.

NACCESS allows the user to choose arbitrary resolution and can therefore be used as a reference for itself, and POPS was optimized with NACCESS as reference. NACCESS uses L&R and performs very similarly to FreeSASA using L&R. The POPS algorithm is intended as a fast coarse-grained approximation; its authors state an average error of 2.6 Å ^2^ per atom ( Cavallo *et al.*, 2003). In Figure 2 the mean *ε* is lower than that, which is expected, since this error is measured over the total SASA, not atom by atom. A fit showed that POPS runs in *O*( *N* ^2^) time (using the data in the file pops.dat in Dataset 2), which to some extent explains the relatively long mean calculation time per atom.

The other programs listed above were left out of Figure 2 because they can not be compared under the same premises. DSSP calculates many different things in addition to its 200 test-point S&R-calculation, and the total running time is therefore naturally longer than the corresponding calculation in FreeSASA, although the accuracy should be comparable for the same number of test points. The program NSOL uses S&R, but does five different SASA calculations on the same input using different parameters. The NSOL code was modified to only do one of the five calculations, and is then only slightly slower than FreeSASA using the same number of test points. Lastly, Triforce is not suitable for comparison in this particular use case because it has a high initialization cost, which makes it slow for calculating the SASA of an isolated structure.

In single-threaded mode, FreeSASA using L&R is almost indistinguishable from NACCESS in Figure 2, but it is significantly faster when 2 or 4 threads are used. The effect of spreading the calculation over several threads is shown in more detail in Figure 3. Since the generation of cell lists is not parallelized, using more than one thread only gives a significant performance benefit in the high resolution limit. Based on these results, the default has been set to two threads. Depending on the nature of the calculations, this speedup can make a noticeable difference.

## Summary

FreeSASA is an efficient library for calculating the SASA of protein, RNA and DNA structures. Its main advantages over other commonly used tools is that it is open source, easily configurable and can be used both as a command line tool, a C library and a Python module. The tests above demonstrate that it runs as fast as, or faster than, some popular tools at a given resolution, and can be boosted further by parallelizing the calculation.

## Data and software availability

### Data

*F1000Research*: Dataset 1. List of PDB codes used for the performance analysis, 10.5256/f1000research.7931.d112977 ( Mitternacht, 2016a).

*F1000Research*: Dataset 2. Zip-archive with raw data for the performance analysis in Figure 2 and Figure 3, 10.5256/f1000research.7931.d112978 ( Mitternacht, 2016b).

### Software

**Latest source code and software available from** http://freesasa.github.io/

**Archived source code as at the time of publication** http://dx.doi.org/10.5281/zenodo.45239 ( Mitternacht, 2016c).

**License** GNU GPL v3 ( http://www.gnu.org/licenses/gpl-3.0.en.html).

## References

[ref-1] Cavallo L Kleinjung J Fraternali F: POPS: A fast algorithm for solvent accessible surface areas at atomic and residue level. *Nucleic Acids Res.* 2003;31(13):3364–3366. 10.1093/nar/gkg601 12824328 PMC169007

[ref-2] DeLano WL: The PyMOL molecular graphics system.2002 Reference Source

[ref-3] Drechsel NJ Fennell CJ Dill KA: TRIFORCE: Tessellated Semianalytical Solvent Exposed Surface Areas and Derivatives. *J Chem Theory Comput.* 2014;10(9):4121–4132. 10.1021/ct5002818 25221446 PMC4159216

[ref-4] Eisenhaber F Lijnzaad P Argos P: The double cubic lattice method: efficient approaches to numerical integration of surface area and volume and to dot surface contouring of molecular assemblies. *J Comput Chem.* 1995;16(3):273–284. 10.1002/jcc.540160303

[ref-5] Finkelstein AV Ptitsyn O: Protein physics: a course of lectures. Academic Press, London,2002 Reference Source

[ref-6] Fraczkiewicz R Braun W: Exact and efficient analytical calculation of the accessible surface areas and their gradients for macromolecules. *J Comput Chem.* 1998;19(3):319–333. 10.1002/(SICI)1096-987X(199802)19:3<319::AID-JCC6>3.0.CO;2-W

[ref-7] Hubbard SJ Thornton JM: NACCESS. *Computer Program, Department of Biochemistry and Molecular Biology*, University College London,1993 Reference Source

[ref-8] Lee B Richards FM: The interpretation of protein structures: estimation of static accessibility. *J Mol Biol.* 1971;55(3):379–400. 10.1016/0022-2836(71)90324-X 5551392

[ref-9] Mantina M Chamberlin AC Valero R: Consistent van der Waals radii for the whole main group. *J Phys Chem A.* 2009;113(19):5806–5812. 10.1021/jp8111556 19382751 PMC3658832

[ref-10] Masuya M: NSOL: A numerical calculation program of molecular surface area, volume, and solvation energy.2003 Reference Source

[ref-11] Mitternacht S: Dataset 1 in: FreeSASA: An open source C library for solvent accessible surface area calculations. *F1000Research.* 2016a Data Source 10.12688/f1000research.7931.1 PMC4776673 26973785

[ref-12] Mitternacht S: Dataset 2 in: FreeSASA: An open source C library for solvent accessible surface area calculations. *F1000Research.* 2016b Data Source 10.12688/f1000research.7931.1 PMC4776673 26973785

[ref-13] Mitternacht S: FreeSASA 1.0.1: Solvent Accessible Surface Area Calculations. *Zenodo.* 2016c Data Source 10.12688/f1000research.7931.1 PMC4776673 26973785

[ref-14] Sanner MF Olson AJ Spehner JC: Reduced surface: an efficient way to compute molecular surfaces. *Biopolymers.* 1996;38(3):305–320. 10.1002/(SICI)1097-0282(199603)38:3<305::AID-BIP4>3.0.CO;2-Y 8906967

[ref-15] Shrake A Rupley JA: Environment and exposure to solvent of protein atoms. Lysozyme and insulin. *J Mol Biol.* 1973;79(2):351–371. 10.1016/0022-2836(73)90011-9 4760134

[ref-16] Swinbank R Purser RJ: Fibonacci grids: A novel approach to global modelling. *Q J R Meteorol Soc.* 2006;132(619):1769–1793. 10.1256/qj.05.227

[ref-17] Touw WG Baakman C Black J: A series of PDB-related databanks for everyday needs. *Nucleic Acids Res.* 2015;43(Database issue):D364–D368. 10.1093/nar/gku1028 25352545 PMC4383885

[ref-18] Tsai J Taylor R Chothia C: The packing density in proteins: standard radii and volumes. *J Mol Biol.* 1999;290(1):253–266. 10.1006/jmbi.1999.2829 10388571

[ref-19] Wang G Dunbrack RL Jr: PISCES: a protein sequence culling server. *Bioinformatics.* 2003;19(12):1589–1591. 10.1093/bioinformatics/btg224 12912846

[ref-20] Weiser J Shenkin PS Still WC: Approximate atomic surfaces from linear combinations of pairwise overlaps (LCPO). *J Comput Chem.* 1999;20(2):217–230. 10.1002/(SICI)1096-987X(19990130)20:2<217::AID-JCC4>3.0.CO;2-A

[ref-21] Westbrook JD Shao C Feng Z: The chemical component dictionary: complete descriptions of constituent molecules in experimentally determined 3D macromolecules in the Protein Data Bank. *Bioinformatics.* 2015;31(8):1274–1278. 10.1093/bioinformatics/btu789 25540181 PMC4393513

[ref-22] Xu D Zhang Y: Generating triangulated macromolecular surfaces by Euclidean Distance Transform. *PLoS One.* 2009;4(12):e8140. 10.1371/journal.pone.0008140 19956577 PMC2779860

