# Supplementary material for: FreeSASA: An open source C library for solvent accessible surface area calculations
Source: F1000Res. 2016 Feb 18;5:189. [Version 1] doi: 10.12688/f1000research.7931.1 (PMC4776673; doi:10.12688/f1000research.7931.1)
Supplement: Peer review history

# Peer review: Referee response for version 1

**DOI:** 10.5256/f1000research.8538.r12528

**Published:** 2016-02-29

**Authors:** Yaoqi Zhou, Yuedong Yang

**Affiliations:** 1 Institute of Glycomics, Griffith University, Gold Coast, QLD, Australia

Solvent accessible surface area is a frequently calculated quantity in structural bioinformatics. Although many tools are available, an open source, easy-to-use program is certainly welcome. The author did a careful test and performed comparative studies to existing tools. The code is clearly written and well documented. There are no major changes required for this clearly written manuscript. Here are some minor questions requiring further clarifications and additions.

1. For multiple chains or a protein-ligand complex structure, does Freesasa yield the ASA for isolated chains or chains in the complex structure? It would be better if the program has an option to calculate the change of ASA on a residue before and after binding to another molecule (ligand, RNA, DNA, another chain) assuming that there is no structural change upon binding. This would allow to identify the functional residues.
2. Please clarify the command to produce a residue-level ASA, rather than the atomic level ASA. 
3. What are default recommendations for the ASA calculation in term of resolution required or choice of LR and SR approximations? 
4. Are the default atomic radii employed here the same as used in DSSP and/or NACCESS? If different, what are the main differences? 
5. Is this method faster than analytical methods? If so, by how much? 
6. Can authors provide a table that lists all possible commands?
7. The program would be more useful if it can directly read in Biopython data structure and work with pymol.

We have read this submission. We believe that we have an appropriate level of expertise to confirm that it is of an acceptable scientific standard.

# Response

**Published:** 2016-02-29

**Authors:** Simon Mitternacht

Thanks for your comments and suggestions. I interpret most of them as being better solved by improving the functionality and/or documentation of the program itself, rather than revising the paper. For the sake of brevity not all the functionality is described in the paper, but is available in the online documentation.

1. The default behavior is to calculate the SASA of the chains in the complex. The command option '--chain-groups' described in the paper allows the user to look at combinations of chains in isolation. The option '--separate-chains', is not discussed in the paper, but can be used to treats each chain separately (i.e. for a four chain protein '--chain-groups=A+B+C+D' would be equivalent to '--separate-chains'). My intention was to give the user the ability to calculate the total ASA of different combinations of chains, and then do the arithmetic of the changes themselves. I will look into adding command-line options to do this automatically in future versions of the program.
2. The option '--foreach-residue' (not described in the paper) can be used to print the SASA of each residue.
3. It is hard to give a general recommendation here, but I chose to use the same default resolution as NACCESS since this seems to be an accepted standard. If one is looking for changes due to minor conformational changes, a higher resolution might be needed. The average of *A*_ref_/ *N* in Dataset 2 is 5.6 Å ^2^, and the average error in Figure 2 for the default resolution is around 0.002 Å ^2^, i.e. the error in the total SASA ( *A*) is on average 0.002/5.6 < 1/1000.
4. As mentioned in the paper, the ProtOr atomic radii are used. I think the easiest way of comparing these with those employed in NACCESS and DSSP is to compare the radii in the configuration-files supplied with the program (in the directory share/).
5. I was not able to get hold of FANTOM or any other analytical program, so I have unfortunately not been able to compare directly. As mentioned I compared with the semi-analytical Triforce, which was significantly slower when run on a single structure due to high initialization cost. I assume Triforce performs better when the same instance of the program is run on many structures, but that is a different use case.
6. The command 'freesasa -h' lists all options. I decided not to provide this list in the paper because it will probably expand as time goes, and then the paper would quickly become outdated.
7. Thank you for these suggestions, I will look into ways of adding more functionality to the Python bindings.

# Peer review: Referee response for version 1

**DOI:** 10.5256/f1000research.8538.r12524

**Published:** 2016-02-26

**Authors:** Simon Hubbard

**Affiliations:** 1 Faculty of Life Sciences, University of Manchester, Manchester, UK

This is a simple and effective implementation of the two main heuristics used for calculating solvent accessible surface area of atomic structures such as proteins. The algortihm, FreeSASA, is suitably described and tested in the article. I see no real need to ask for any changes, as the author has done a very professional and nice job here. It downloads and installs very smoothly and offers most of the functionality that NACCESS offers, so will be appreciated by legacy users I would imagine (though some things have not been implemented, probably with good reason - either way, not a problem for me - and the author can react to requests or users can do a little work to get what they want in terms of formatting). I am sure the ability to thread across multiple cores will be beneficial and speed things up, and the API will make it slot into python pipelines etc. It's possible that it won't directly supplant some NACCESS dependencies which rely on its rather old formatting (.rsa file for example perhaps) but this is probably only a minor concern and easily fixed if needed (since its fully open source, its probably reduced to changing a few print statements anyway).

I have read this submission. I believe that I have an appropriate level of expertise to confirm that it is of an acceptable scientific standard.
